# Prognostic factor analysis of definitive radiotherapy using intensity-modulated radiation therapy and volumetric modulated arc therapy with boluses for scalp angiosarcomas

**DOI:** 10.1038/s41598-022-08362-2

**Published:** 2022-03-14

**Authors:** Tairo Kashihara, Hiroshi Igaki, Dai Ogata, Hiroki Nakayama, Satoshi Nakamura, Kae Okuma, Taisuke Mori, Kohei Yamakawa, Akira Takahashi, Kenjiro Namikawa, Ayaka Takahashi, Kana Takahashi, Tomoya Kaneda, Koji Inaba, Naoya Murakami, Yuko Nakayama, Hiroyuki Okamoto, Naoya Yamazaki, Jun Itami

**Affiliations:** 1 Department of Radiation Oncology, National Cancer Center Hospital, 5-1-1 Tsukiji, Chuo-ku, Tokyo, 104-0045 Japan; 2 Department of Dermatologic Oncology, National Cancer Center Hospital, 5-1-1 Tsukiji, Chuo-ku, Tokyo, 104-0045 Japan; 3 Department of Pathology and Clinical Laboratories, National Cancer Center Hospital, 5-1-1 Tsukiji, Chuo-ku, Tokyo, 104-0045 Japan

**Keywords:** Oncology, Risk factors, Outcomes research, Radiotherapy

## Abstract

Cutaneous angiosarcomas is a rare cancer with poor prognoses. The common radiotherapy techniques that have been reported so far are two pairs of lateral X-ray and electron fields. However, it is quite difficult to irradiate scalp angiosarcomas (SAs) homogeneously with this technique. In this study, safety, effectiveness, and risk factors were assessed for localized SAs ≥ 5 cm treated with intensity-modulated radiotherapy (IMRT) or volumetric modulated arc therapy (VMAT) with boluses. Sixty-eight angiosarcoma patients who had received radiotherapy in our institution between January 2007 and November 2020 were retrieved from our radiotherapy database. Of these patients, 27 localized SA patients were included in the retrospective analysis. The 2-year overall survival, local progression-free rate, and distant metastases-free survival were 41.8%, 48.4%, and 33.1%. All the patients experienced acute radiation dermatitis ≥ grade 2, with18 (66.7%) ≥ grade 3. No nodule lesion was a significant unfavorable predictive factor of acute radiation dermatitis ≥ grade 3. Tumor bleeding at the initiation of radiotherapy and tumor invasion to the face were significant predictive factors of overall survival, and tumor bleeding at the initiation of radiotherapy was also a significant predictive factor of local progression-free rate.

## Introduction

Cutaneous angiosarcoma is a rare cancer with a poor prognosis and often appears on the scalp and face^1–6^. Although aggressive surgical resection is associated with better prognosis^7,8^. radiation therapy (RT) without surgical resection is one of the treatment options. In particular, chemoradiotherapy with taxane was reported to be superior to surgery and RT in a multicenter retrospective study^9^. The most common RT technique that has been reported so far for scalp angiosarcomas is the conventional method of combining two pairs of lateral X-ray and electron fields^10–12^. However, it is quite difficult to irradiate scalp angiosarcomas homogeneously with this technique because scalps are spherical, and it is especially difficult when the tumors are widespread. On the other hand, RT techniques have been evolving, and intensity-modulated radiation therapy (IMRT) and volumetric modulated arc therapy (VMAT) have been reported to have fewer adverse effects and may enhance the effectiveness by dose escalation using simultaneous-integrated boost technique in head and neck cancer^13–17^. Nevertheless, there are few reports of the scalp angiosarcoma patients treated with IMRT or VMAT without surgery. In Bernstein et al.^18^, IMRT was used for 21 patients; however, there were a few patients in that study who received definitive radiotherapy without surgery, and the remaining patients were treated with surgery alone or surgery with postoperative RT.

In our institution, IMRT or VMAT has been used to irradiate scalp angiosarcomas ≥ 5 cm to irradiate the tumors homogeneously. Herein, we retrospectively investigated the safety and effectiveness of definitive RT with IMRT or VMAT for localized scalp angiosarcomas ≥ 5 cm by excluding patients who received postoperative RT.

## Materials and methods

### Patient population

Sixty-eight angiosarcoma patients who had received RT at our institution between January 2007 and November 2020 were retrieved from our RT database. Of these patients, 22 received postoperative RT to prevent recurrence, 13 had nodal or distant metastases, 1 was an angiosarcoma of the lower leg and a face, 2 received high-dose-rate brachytherapy using the multi-catheter mold technique, and 2 received only electron beam RT. In total, 27 localized scalp angiosarcoma patients were included in the retrospective analysis. Positron emission tomography/computed tomography (CT) was taken only in 7 patients, but CT of the head to pelvis was taken in all patients prior to treatment to rule out metastases. A flowchart of the patient selection is shown in Supplementary Figure 1. The following factors were investigated: age, sex, Zubrod performance status, single life, albumin level at the initiation of RT, tumor invasion to the face, postoperative recurrence, concurrent administration of chemotherapy, adjuvant chemotherapy, prescription radiation dose, prescription method, and clinical target volume (CTV). The face was defined as the area below the eyebrows.

### Treatment methods

To accurately identify the extent of the lesions, all the patients had their heads shaved prior to RT. The treatment planning CT images were taken with markers attached to the skin by dermatologists and radiation oncologists as a marker for the CTV, with a 5–10 mm bolus (Supplementary Figure 2) and an immobilization mask. In general, the CTV ranged from 2 to 4 cm radially along the scalp from the edge of the gross tumor volume. The whole scalp was not defined as CTV. The planning target volume (PTV) margins were set to 3–5 mm in all directions. When the PTV extended outside the body, it was cropped to the body surface. No patient was irradiated to the cervical lymph nodes. The slice thickness of the treatment planning CT images was 2 mm. Twenty-five patients were treated with VMAT and the other two were treated with IMRT using the step and shoot technique. In three patients, intentional internal high-dose policy (IIHDP) VMAT was applied for prominent tumor lesions. The prescription dose was 51–70 Gy at 2–3 Gy per fraction, once a day and five times per week. RT was delivered with 4 MV X-rays from linear accelerators (Varian, Palo Alto, CA, USA). The prescription dose was introduced to 50–95% of the PTV or planned to be the mean dose of PTV.

### Assessment of outcomes

Overall survival (OS), cause-specific survival (CSS), disease-free survival (DFS), local progression-free rate (LPFR), locoregional progression-free rate (LRPFR), and distant metastasis-free survival (DMFS) were investigated as treatment outcomes. Local recurrence was defined as recurrence inside the PTV, and locoregional recurrence was defined as recurrence inside or at the outer edge of the PTV. Deaths other than deaths from angiosarcoma were treated as censored in CSS. Dermatitis, mucositis, and other adverse effects ≥ grade 2 due to RT were investigated as acute side effects. Furthermore, skin disorders, mucosal damage, and other adverse effects ≥ grade 2 were investigated as late side effects. Side effects were evaluated using the Common Terminology Criteria for Adverse Events (CTCAE) ver. 5.0. Acute side effects were defined as those that appeared within 3 months of the end of radiotherapy, and late side effects were defined as those that appeared after that.

### Statistical analyses

Predictive factors of radiation dermatitis ≥ grade 3 were estimated using logistic regression analysis. The 2-year OS, CSS, DFS, LPFR, LRPFR, DMFS, median OS, and DMFS were estimated using the Kaplan–Meier method. The predictive factors of OS, LPFR, and DMFS were investigated using Cox proportional hazards regression models. The median value was used to divide the patients into two groups. Factors with *p* values < 0.1 in the univariate analyses were included in the multivariate analyses. *p* values < 0.05 were considered statistically significant in the multivariate analyses. All the statistical analyses were performed using IBM SPSS version 26 software (IBM Corp., Armonk, NY, USA).

### Ethical approval

For all research involving human participants/data, written informed consent to participate in the study was obtained from participants. Informed consent for publication of images was also obtained from the participants or legally authorized representatives. All analyses involving human participants performed in this study were approved by the institutional review board of the committee in National Cancer Center Hospital (approval number, 2017-091) and were in accordance with the ethical standards of the committee and with the 1964 Helsinki Declaration and its later amendments.

## Results

### Patient/treatment characteristics

The patient and treatment characteristics are shown in Table 1. The median age was 76 years (range 46–89). The median size of the lesions was 17.9 cm (range 7.8–23.1 cm). The median RT duration was 52 days (range 25–70). The most commonly used prescription radiation dose was 70 Gy in 35 fractions (77.8%). Two patients were treated with a combination of electron beams and X-rays: one received irradiation using electron beams to the area around the eyes with lead contact lenses, and in the other patient, electron beams were used to initiate RT as soon as possible. Twenty-two patients (81.5%) received concurrent chemoradiotherapy. The regimen was weekly paclitaxel in all the patients. Five patients were treated for postoperative recurrence lesions.

**Table 1:** Patient and treatment characteristics.

Parameters	
Age, median (range)	76 (46–89)
**Sex**	
Male	6/27 (22.2%)
Female	21/27 (77.8%)
**Zubrod performance status**	
0	14/27 (51.9%)
1	11/27 (40.7%)
2	2/27 (7.4%)
**Single life**	
Yes	3/27 (11.1%)
No	24/27 (88.9%)
Albumin level at the initiation of radiation therapy, median (range)	4.0 (3.2–4.8)
**Tumor invasion to face**	
Yes	11/27 (40.7%)
No	16/27 (59.3%)
**With tumor on the posterior part of the auricle at the initiation of radiation therapy**	
Yes	9/27 (33.3%)
No	18/27 (66.7%)
**Tumor bleeding at the initiation of radiation therapy**	
Yes	7/27 (25.9%)
No	20/27 (74.1%)
**Skip lesions**	
Yes	23/27 (85.2%)
No	4/27 (14.8%)
**With nodule lesions**	
Yes	10/27 (37.0%)
No	17/27 (63.0%)
**With ulcerative lesions**	
Yes	3/27 (11.1%)
No	24/27 (88.9%)
Postoperative recurrence	5/27 (18.5%)
**Concurrent administration of chemotherapy**	
Yes, number	22/27 (81.5%)
No, number	5/27 (18.5%)
**Adjuvant chemotherapy**	
Yes	8/27 (28.6%)
No	19/27 (71.4%)
**Prescription radiation dose**	
70 Gy in 35 fractions	21/27 (77.8%)
66 Gy in 33 fractions	1/27 (3.7%)
60 Gy in 30 fractions	3/27 (11.1%)
66 Gy in 22 fractions	1/27 (3.7%)
51 Gy in 17 fractions	1/27 (3.7%)
**Prescription method**	
**Delivered prescription dose**	
To 50% of the PTV*	3/27 (11.1%)
To 70% of the PTV	1/27 (3.7%)
To 90% of the PTV	3/27 (11.1%)
To 95% of the PTV	14/27 (51.9%)
Equal to the mean dose of PTV	6/27 (22.2%)
Clinical target volume (cc), median (range)	267.7 (68.5–694.3)

PTV*, planning target volume.

### Adverse effects and survival analysis

**Figure 1:**
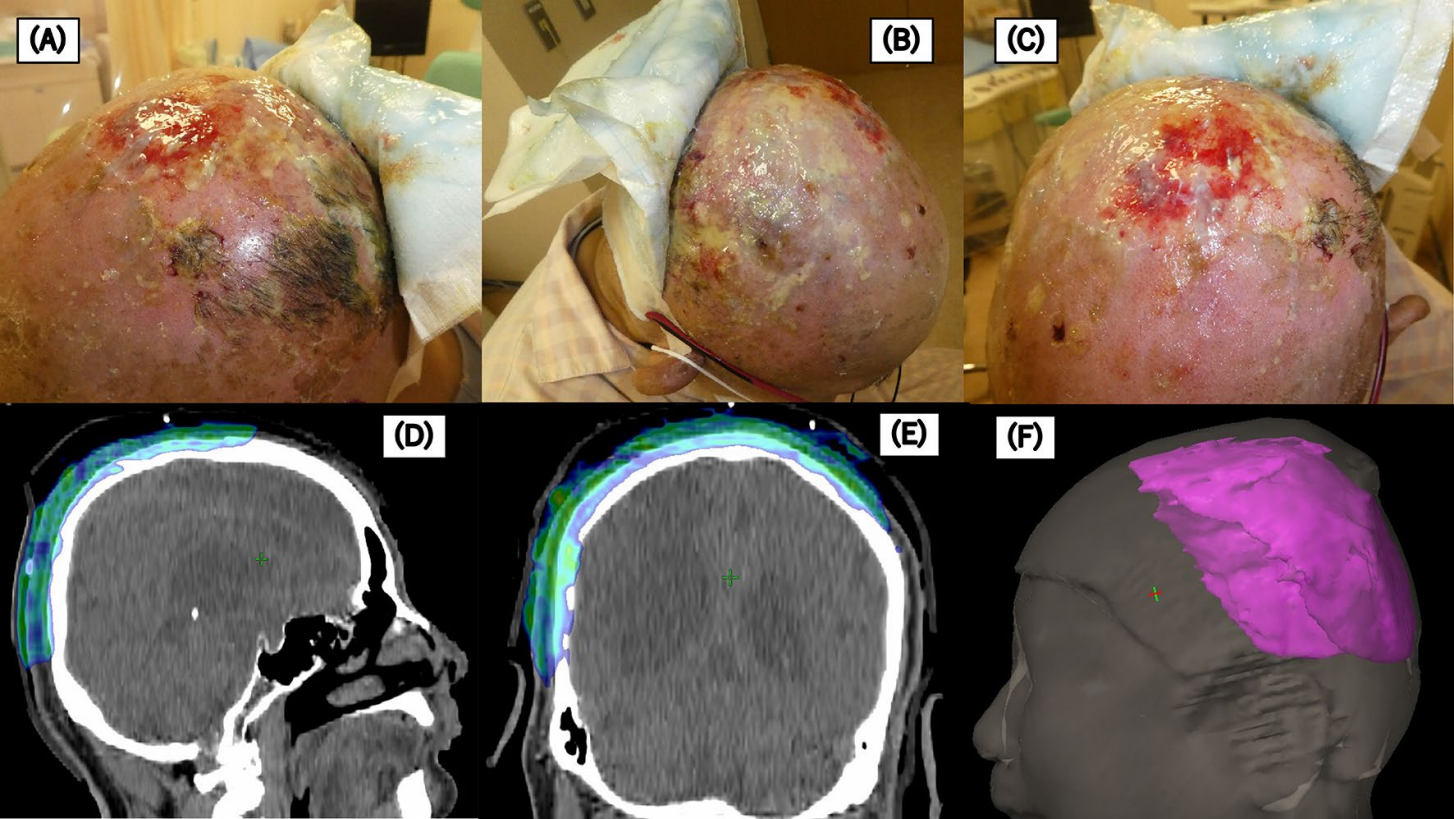
A case of a patient with severe dermatitis and the dose distribution. In figure (**D**,**E**), the region where 100% dose of the prescribed dose was irradiated is shown by dose-color-wash. In figure (**F**), the region where 100% dose of the prescribed dose was irradiated is displayed in three dimensions.

The median follow-up period was 9 months (range 4–39 months). The acute and late side effects associated with RT are shown in Supplementary Table 1. All the patients experienced acute radiation dermatitis ≥ grade 2, and 18 patients (66.7%) had acute radiation dermatitis ≥ grade 3. Images of patients who experienced severe radiation dermatitis and the dose distribution are shown in Fig. 1. Acute mucositis ≥ grade 2 was detected in 6 patients (22.2%). RT was suspended for a week in two patients due to severe acute radiation dermatitis and mucositis, respectively. In addition, the total radiation dose was reduced in two patients due to severe radiation dermatitis and delirium associated with the infection from severe mucositis. There was one case of grade 3 acute conjunctivitis and keratitis, and one case each of grade 3 skin ulceration, grade 3 keratitis, and grade 3 ophthalmalgia as late side effects. Only the factor of no nodule lesions was associated with acute radiation dermatitis ≥ grade 3 (p = 0.032, Table 2).

**Table 2:** Analyses of predictive factors on acute radiation dermatitis ≥ grade 3.

Parameters	n (%)	*p* value
**Age ≥ 76**		*p* = 0.272
Yes	8/14 (57.1%)	
No	10/13 (76.9%)	
**Sex**		*p* = 0.326
Men	13/21 (61.9%)	
Women	5/6 (83.3%)	
**Zubrod performance status**		*p* = 0.173
0	11/14 (78.6%)	
1 or 2	7/13 (53.8%)	
**Single life**		*p* = 0.194
Yes	1/3 (33.3%)	
No	17/24 (70.8%)	
**Albumin level ≥ 4 at the initiation of radiation therapy**		*p* = 0.785
Yes	9/14 (64.3%)	
No	9/13 (69.2%)	
**Tumor invasion to face**		*p* = 0.782
Yes	7/11 (63.6%)	
No	11/16 (68.8%)	
**With tumor on the posterior part of the auricle at the initiation of radiation therapy**		*p* = 1.000
Yes	6/9 (66.7%)	
No	12/18 (66.7%)	
**Tumor bleeding at the initiation of radiation therapy**		*p* = 0.214
Yes	6/7 (85.7%)	
No	12/20 (60.0%)	
**Skip lesions**		*p* = 0.702
Yes	15/23 (65.2%)	
No	3/4 (75.0%)	
**With nodule lesions**		*p* = 0.032
Yes	4/10 (40.0%)	
No	14/17 (82.4%)	
**With ulcerative lesions**		*p* = 0.194
Yes	3/3 (100.0%)	
No	15/24 (62.5%)	
**Concurrent administration of chemotherapy**		*p* = 0.726
Yes	15/22 (68.2%)	
No	3/5 (60.0%)	
**Adjuvant chemotherapy**		*p* = 0.766
Yes	5/8 (62.5%)	
No	13/19 (5.3%)	
**Total biological effective dose**		*p* = 0.636
< 80 Gy	4/5 (80.0%)	
≥ 80 Gy	14/22 (63.6%)	
**Prescription method**		
**Delivered prescription dose to less than 95% of the PTV**		*p* = 0.276
Yes	8/14 (57.1%)	
No	10/13 (76.9%)	
**Clinical target volume**		*p* = 0.173
< 260 cc	7/13 (53.8%)	
≥ 260 cc	11/14 (78.6%)	

PTV*, planning target volume.

The 2-year OS, CSS, DFS, LPFR, LRPFR and DMFS were 41.8%, 45.5%, 29.8%, 48.4%, 40.5%, and 33.1%, respectively. The median OS and DMFS were 9 months (range 4–39 months) and 8 months (range 3–31 months). Local recurrences were observed in 12 patients (44.4%). Of these, three recurrences were detected in the entire lesions, four were in prominent lesions, and one was in the largest lesion. Of the other four flat lesions, two were only on the posterior part of the auricle, one was only a forehead lesion, and one was a sinciput. In one of the four patients who experienced recurrences in prominent lesions, the flat and small lesion on the posterior part of the auricle also recurred. Additionally, two of the three patients who experienced recurrences in the entire lesion also had recurrence on the posterior part of the auricle. In total, five flat lesions on the posterior part of the auricle recurred. Distant metastases were detected in 12 patients (44.4%), and the median time from the initiation of RT to the appearance of local recurrences and distant metastases was 3 months (range 2–25 months) and 9 months (range 3–26 months), respectively.

**Figure 2:**
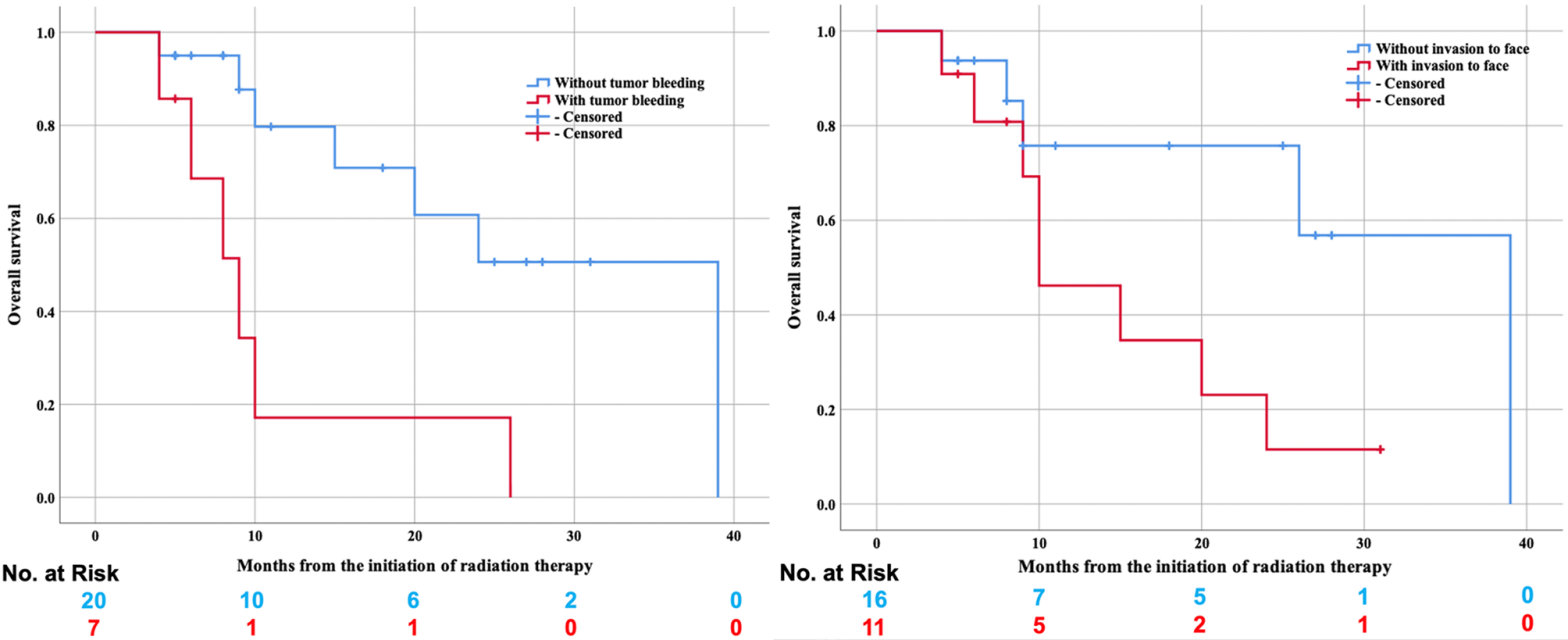
The Kaplan–Meier curves of OS* in accordance with tumor bleeding at the initiation of radiation therapy and tumor invasion to the face. OS*, overall survival.

In univariate analyses with Cox proportional hazards regression models of OS and DMFS, tumor bleeding at the initiation of RT and CTV ≥ 260 cc were significant predictive factors (Table 3). Furthermore, tumor bleeding at the initiation of RT, total biological effective dose (BED) < 80 Gy based on the linear-quadratic model with the α/β ratio of 10 Gy and tumor invasion to the face were unfavorable predictive factors of LPFR, and tumor invasion to the face was an unfavorable factor of OS with marginal statistical significance. In multivariate analyses, tumor bleeding at the initiation of RT was a significant predictive factor of OS and LPFR, and tumor invasion to the face was also a significant predictive factor of OS (Table 3, Fig. 2).

**Table 3:** The univariate and multivariate analyses of OS, LPFR, and DMFS.

Parameters		
OS	LPFR	DMFS
HR (95%CI), *p* value	HR (95%CI), *p* value	HR (95%CI), *p* value
**Univariate analyses**		
Age ≥ 76		
0.898 (0.288–2.807), *p* = 0.854	0.562 (0.176–1.790), *p* = 0.329	0.824 (0.288–2.357), *p* = 0.719
Sex (vs female)		
0.800 (0.209–3.060), *p* = 0.749	1.126 (0.242–5.235), *p* = 0.880	0.601 (0.176–2.059), *p* = 0.418
Zubrod performance status (vs 1–2)		
0.861 (0.272–2.722), *p* = 0.798	0.766 (0.240–2.444), *p* = 0.653	0.592 (0.198–1.769), *p* = 0.348
Single life		
1.023 (0.220–4.752), *p* = 0.977	0.488 (0.062–3.853), *p* = 0.496	0.869 (0.189–4.003), *p* = 0.857
Albumin level ≥ 4 at the initiation of radiation therapy		
0.571 (0.171–1.911), *p* = 0.363	1.156 (0.368–3.629), *p* = 0.804	0.468 (0.146–1.495), *p* = 0.200
Tumor invasion to face		
**3.179 (0.940–10.747), *p* = 0.063**	**2.874 (0.900–9.181), *p* = 0.075**	2.361 (0.804–6.939), *p* = 0.118
With tumor on the posterior part of the auricle at the initiation of radiation therapy		
1.679 (0.530–5.314), *p* = 0.378	1.966 (0.616–6.273), *p* = 0.254	1.521 (0.502–4.608), *p* = 0.459
Tumor bleeding at the initiation of radiation therapy		
**4.571 (1.450–14.405), *p* = 0.009**	**2.812 (0.882–8.967), *p* = 0.081**	**4.411, (1.446–13.457), *p* = 0.009**
Skip lesions		
0.440 (0.092–2.100), *p* = 0.303	0.515 (0.106–2.495), *p* = 0.410	0.561 (0.122–2.582), *p* = 0.458
Nodule lesions		
1.620 (0.494–5.316), *p* = 0.426	1.275 (0.386–4.214), *p* = 0.690	1.778 (0.593–5.333), *p* = 0.304
Ulceration lesions		
1.487 (0.304–7.280), *p* = 0.624	0.837 (0.103–6.315), *p* = 0.837	1.272 (0.273–5.918), *p* = 0.759
Concurrent administration of chemotherapy		
0.642 (0.168–2.454), *p* = 0.517	1.110 (0.238–5.180), *p* = 0.894	0.947 (0.258–3.481), *p* = 0.935
Adjuvant chemotherapy		
0.385 (0.100–1.483), *p* = 0.166	0.941 (0.277–3.197), *p* = 0.923,	0.432 (0.130–1.432), *p* = 0.170
Total biological effective dose ≥ 80 Gy		
0.507 (0.136–1.886), *p* = 0.311	**0.342 (0.101–1.165), *p* = 0.086**	0.386 (0.1118–1.257), *p* = 0.114
Prescription method (delivered prescription dose to less than 95% of the PTV)		
1.909 (0.508–7.172), *p* = 0.338	1.293 (0.399–4.187), *p* = 0.668	1.729 (0.536–5.575), *p* = 0.359
Clinical target volume ≥ 260 cc		
**4.347 (1.163–16.253), *p* = 0.029**	2.467 (0.723–8.414), *p* = 0.149	**3.921 (1.213–12.676), *p*** = **0.022**
**Multivariate analyses**		
Tumor invasion to face		
**4.641 (1.189–18.117), *p* = 0.027**	3.700 (0.970–14.116), *p* = 0.056	
Tumor bleeding at the initiation of radiation therapy		
**4.365 (1.027–18.553), *p* = 0.046**	3.984 (1.093–14.524)**, *p* = 0.036**	2.519 (0.691–9.180), *p* = 0.162
Total biological effective dose ≥ 80 Gy		
	0.479 (0.115–1.999), *p* = 0.313	
Clinical target volume ≥ 260 cc		
2.129 (0.416–10.889), *p* = 0.364	2.584 (0.650–10.266), *p* = 0.177	

OS*, overall survival, LPFR^†^; local progression-free rate, DMFS^‡^; distant metastases-free survival.

## Discussion

In the present study, we investigated the safety and effectiveness of IMRT or VMAT for localized scalp angiosarcomas ≥ 5 cm. No nodule lesion was a significant unfavorable predictive factor of acute radiation dermatitis ≥ grade 3. In addition, tumor bleeding at the initiation of RT and tumor invasion to the face were significant predictive factors of OS, and tumor bleeding at the initiation of RT was also a significant predictive factor of LPFR.

Scalp angiosarcomas are treated with multiple modalities, including surgery, chemotherapy, and RT. There are some reports on the outcomes of definitive RT with or without chemotherapy without surgery. Hata et al. reported on 17 scalp angiosarcoma patients who received definitive radiotherapy without surgery^11^. In this study, total scalp irradiation was performed using the conventional method of combining X-rays and electron beams. There were 9 patients with tumors < 5 cm, and the 3-year local control rate and OS were 86% and 23%, respectively. All the patients experienced grade 1 or 2 radiation dermatitis, but none developed side effects ≥ grade 3. In our study, 18 of 27 patients (66.7%) had radiation dermatitis ≥ grade 3. Inter- or intra-fractional movement of the bolus and the patient may also be a major factor in the severe dermatitis. When the PTV extends close to the skin, IMRT and VMAT can boost the fluence from tangential angles to superficial parts of the PTV due to the buildup effect^19^. In addition, an analysis of predictive factors of acute radiation dermatitis ≥ grade 3 revealed that having a nodular lesion was a significant favorable predictive factor in our study, which may be because normal skin has already been destroyed in nodular lesions and dermatitis cannot occur. On the other hand, local recurrence was higher in our report compared to that by Hata et al. Local recurrences were detected in 5 prominent or larger lesions. All the patients in our study had tumors ≥ 5 cm, and our patients might have larger lesions than those in this preceding report. Furthermore, Suzuki et al. reported that a total BED ≥ 95 Gy was associated with improved local control^20^. In our study, total biological effective dose ≥ 80 Gy tended to have a higher LPFS (OR 0.342, *p* = 0.086) as shown in Table 3. It may be useful to analyze the results by dose–volume histogram (DVH) assessment instead of prescription dose. However, we did not analyze the correlation of dermatitis and treatment outcomes with DVH on the treatment planning CT because the lack of daily reproducibility of the bolus position is a major problem in our study, thus it is not likely to reflect the real situation and it could lead to wrong conclusions. On the other hand, no patients received a total BED ≥ 95 Gy in our patient group, and the low BED might also be a reason for the higher rate of local progression. Nevertheless, there were many patients in the present study with severe radiation dermatitis; thus, it was difficult to escalate the total radiation dose. Meanwhile, IIHDP VMAT was applied for prominent lesions in 3 patients in our study. IIHDP RT is a technique that increases the radiation dose only inside the tumor while protecting organs at risk (OARs)^17,21^. No local recurrences were detected in those three lesions. This technique might improve local control of prominent lesions without increasing the risk of severe radiation dermatitis. For flat lesions, other modalities such as boron neutron capture therapy, which is currently being tested for localized angiosarcoma in a clinical trial in Japan, might be a breakthrough in the treatment of scalp angiosarcomas by killing cancer cells while sparing normal cells.

There was a trend for more local recurrence, and OS was significantly worse in the patients with facial invasions of tumors in our study. Tumor invasion to the face could inevitably decrease the irradiated dose considering the dose constraints to OARs of the face, such as the eyes or ears. Dose de-escalation might lead to local progression of tumors on the face, although Bernstein et al. reported that patients with face angiosarcomas had better survival than scalp angiosarcomas^18^. Nonetheless, most of the patients in this preceding report received surgery, and scalp angiosarcoma patients had 27% larger tumors. In fact, there were 5 cases of acute conjunctivitis ≥ grade 2, 3 of keratitis ≥ grade 2, 2 middle ear infections ≥ grade 2, and 1 case of late grade 3 keratitis in our study. Thus, cutaneous angiosarcoma close to the eyes or ears may be difficult to cure without side effects with definitive RT without surgery. Furthermore, there were five recurrences in the posterior part of the auricle, although they were flat lesions at the initiation of RT. This may be due to the difficulty in reproducibility of the dose distribution because it is difficult to ensure reproducibility of the position of the bolus behind the ear. In our institution, the bolus is customized by radiological technicians in accordance with the extent of the scalp lesion (Supplementary Figure 2) and cut out to fit the shape of the head when the treatment planning CT is taken, but it is difficult to exactly fit the shape of the head. Three-dimensional printed boluses tailored to individual patients may solve this problem.

To the best of our knowledge, this is the largest report on clinical results and predictive factors on treatment outcomes and severe dermatitis of IMRT or VMAT using bolus. There are few reports of scalp angiosarcomas treated with IMRT or VMAT because electron beams and three-dimensional conformal RT (3DCRT) were utilized in most of the studies on scalp angiosarcomas, therefore our study would be one of the few valuable reports using IMRT or VMAT. We excluded patients who were treated with postoperative RT, palliative RT, or RT with electron beams or 3DCRT alone, and those who had nodal or distant metastases, and thus analyzed a uniform patient group.

However, there are three limitations in this study. First, in this study, IMRT and VMAT were performed using 4MV, and the results may be different when 6MV is used. Second, a systematic review and meta-analysis reported that definitive surgical resection was associated with an improvement in OS^22^. Patients who underwent surgery were excluded from this study, and future analyses should include such patients. Third, this was a retrospective study with a small number of patients and a short follow-up period; therefore, it could include some unknown biases. Further studies are needed to investigate the role of IMRT and VMAT.

## Conclusion

In the patients with localized scalp angiosarcomas ≥ 5 cm treated with IMRT or VMAT with boluses, tumor bleeding at the initiation of RT and tumor invasion to the face were significant predictive factors of OS, and tumor bleeding at the initiation of RT was also a significant predictive factor of LPFR. In addition, no nodule lesion was a significant unfavorable predictive factor of acute radiation dermatitis ≥ grade 3.

## Supplementary Information

Supplementary Figure 1.

Supplementary Figure 2.

Supplementary Table 1.

## Data Availability

The data that support the findings of this study are available from the corresponding author upon reasonable request.

## Abbreviations

RT: Radiation therapy
IMRT: Intensity-modulated radiation therapy
VMAT: Volumetric-modulated arc therapy
CTV: Clinical target volume
PTV: Planning target volume
IIHDP: Intentional internal high-dose policy
OS: Overall survival
CSS: Cause-specific survival
DFS: Disease-free survival
LPFR: Local progression-free rate
LRPFR: Locoregional progression-free rate
DMFS: Distant metastasis-free rate
OAR: Organ at risk
3DCRT: Three-dimensional conformal radiation therapy
