# Supplementary figures and images for: Prognostic factor analysis of definitive radiotherapy using intensity-modulated radiation therapy and volumetric modulated arc therapy with boluses for scalp angiosarcomas
Source: Sci Rep. 2022 Mar 14;12:4355. doi: 10.1038/s41598-022-08362-2 (PMC8921322; doi:10.1038/s41598-022-08362-2)

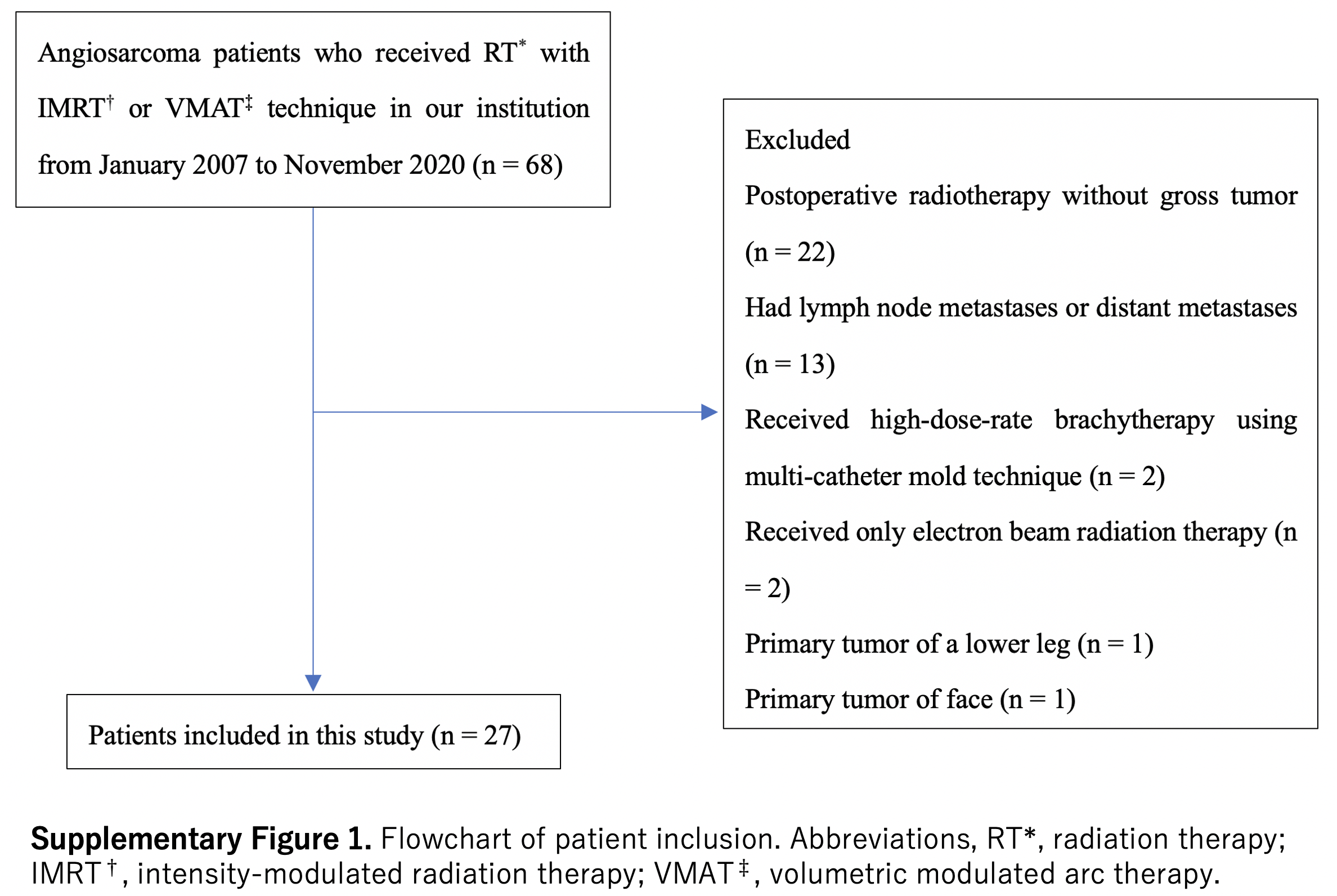

Supplement: Supplementary file 1 — Supplementary Figure 1. [file 41598_2022_8362_MOESM1_ESM.png]

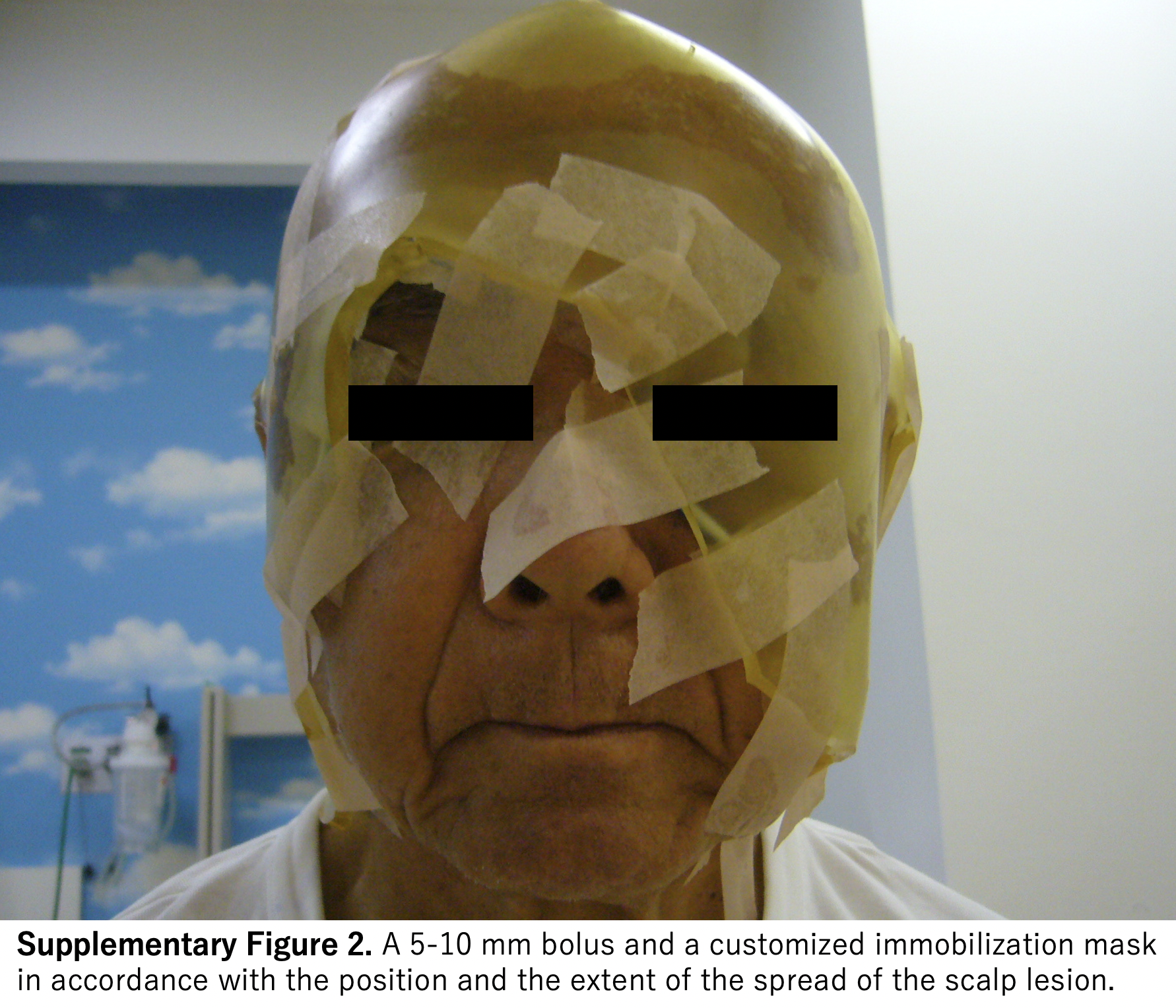

Supplement: Supplementary file 2 — Supplementary Figure 2. [file 41598_2022_8362_MOESM2_ESM.png]
