# Supplementary material for: Prognostic factor analysis of definitive radiotherapy using intensity-modulated radiation therapy and volumetric modulated arc therapy with boluses for scalp angiosarcomas
Source: Sci Rep. 2022 Mar 14;12:4355. doi: 10.1038/s41598-022-08362-2 (PMC8921322; doi:10.1038/s41598-022-08362-2)
Supplement: Supplementary file 3 — Supplementary Table 1. [file 41598_2022_8362_MOESM3_ESM.docx]

Supplementary Table 1. Acute and late adverse effects ≥ grade 2.

Parameters CTCAE grade n (%)


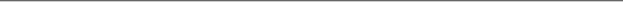


**Acute adverse effects**

Dermatitis

grade 2 9/27 (33.3%)

grade 3 13/27 (48.1%)

grade 4 5/27 (18.5%)

Mucositis

grade 2 2/27 (7.4%)

grade 3 3/27 (11.1%)

grade 4 1/27 (3.7%)

Conjunctivitis

grade 2 4/27 (14.8%)

grade 3 1/27 (3.7%)

*Keratitis*

grade 2 2/27 (7.4%)

grade 3 1/27 (3.7%)

*Dry eye*

grade 2 4/27 (14.8%)

Nasal obstruction

grade 2 1/27 (3.7%)
Middle ear infection

grade 2 2/27 (7.4%)

*Dry mouth*

grade 2 3/27 (11.1%)

Dysgeusia

grade 2 6/27 (22.2%)

Delirium

grade 3 1/27 (3.7%)


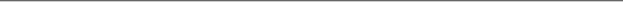


**Late adverse effects**

Skin ulcer

grade 2 1/27 (3.7%)

grade 3 1/27 (3.7%)

*Keratitis*

grade 3 1/27 (3.7%)

Hearing impairment

grade 2 1/27 (3.7%)

Ophthalmalgia

grade 3 1/27 (3.7%)

*Dry mouth*

grade 2 1/27 (3.7%)

Dysgeusia

grade 2 1/27 (3.7%)


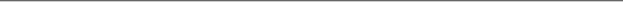


CTCAE^*^, Common Terminology Criteria for Adverse Events
